# Vibration and Fluorescence Spectra of Porphyrin-Cored 2,2-Bis(methylol)-propionic Acid Dendrimers

**DOI:** 10.3390/s90301937

**Published:** 2009-03-16

**Authors:** Boris Minaev, Mikael Lindgren

**Affiliations:** 1 Department of Chemistry, Bogdan Hmelnitskij National University, 18031 Cherkassy, Ukraine; E-Mail: bfmin@rambler.ru; 2 Department of Physics, Norwegian University of Science and Technology, 7491 Trondheim, Norway

**Keywords:** Porphyrin dendrimers, fluorescence spectroscopy, IR spectroscopy, time-resolved spectroscopy, dendrimer size effect

## Abstract

Bis-MPA dendron-coated free-base tetraphenylporphyrin and zinc-tetraphenyl-porphyrin (TPPH_2_ and TPPZn) were studied in comparison with simple porphyrins (H_2_P, ZnP) by theoretical simulation of their infrared, Raman and electronic absorption spectra, as well as fluorescense emission. Infrared and fluorescence spectra of the dendrimers were measured and interpreted along with time-resolved measurements of the fluorescence. The 0–1 emission band of the dendron substituted TPPZn was found to experience a “heavy substitution”-effect. The 0–1 vibronic emission signal is associated with a longer decay time (approx. 7 - 8 ns) than the 0-0 emission (approx. 1 - 1.5 ns). The former contributed with more relative emission yield for larger dendron substituents, in agreement with the appearance of steady-state emission spectra showing increased contribution from the 0–1 vibronic fluorescence band at 650 nm. No such substitution effect was observed in the electronic or vibrational spectra of the substituted free-base variant, TPPH_2_. Vibration spectra of the parent porphyrins (H_2_P, ZnP, TPPH_2_ and TPPZn) were calculated by density functional theory (DFT) using the B3LYP/6-31G** approximation and a detailed analysis of the most active vibration modes was made based on both literature and our own experimental data. Based on the results of theoretical calculations the wide vibronic bands in the visible region were assigned. The vibronic structure also gave a qualitative interpretation of bands in the electronic absorption spectra as well as in fluorescence emission depending on the size of dendrimer substitution. From the results of time-dependent DFT calculations it is suggested that the TPPZn-cored dendrimers indicate strong vibronic interaction and increased Jahn-Teller distortion of the prophyrin core for larger dendrimer generations. Specifically, this leads to the entirely different behaviour of the emission spectra upon substitution of the TPPH_2_ and TPPZn variants, which was also experimentally observed. Since TPPH_2_ is originally of lower symmetry the specific distortion upon dendron substitution is not expected to the same extent, which also was in agreement with the experimental findings.

## 1. Introduction

Porphyrins are important chromophores that play a crucial role in a number of biological processes such as photosynthesis, dioxygen transport and activation, and photodynamic cancer therapy [1–4]. The study of excited states of porphyrins is important for the understanding of their electronic structure in the context of various applications. Porphyrin photochemistry also provides insight into the dynamics of related biomolecules, such as the photosynthetic reaction centers in purple bacteria and green plants and heme-based metalloproteins such as hemoglobin and myoglobin. Much of this work has recently been focused on free-base and metalloporphyrin assemblies for light-harvesting purposes, porphyrin containing mimics of the photosynthetic reaction center, and electronic devices. The last decades have witnessed a vast number of experimental studies of porphyrins which have yielded very useful information about their electronic structure and optical spectra (see for example, [1–3,5–7]), but it has not always been possible to provide a well reasoned explanation of the results obtained [8–12]. Although the absorption and fluorescence spectra of many porphyrins are well-known [13–15], the vibronic band structures are not completely understood so far, apart for the fundamental free-base porphyrin that recently was interpreted on the basis of rigorous theoretical investigations [16,17].

Recently, the harmonic vibrational frequencies of a number of porphyrins (H_2_P, ZnP, MgP) and vibronic intensities in phosphorescence, in the first absorption (*Q_x_*) and fluorescence bands were investigated by density functional theory (DFT) [18], also taking vibronic perturbations into account [16,17]. The transition probability was calculated by time-dependent DFT with Franck-Condon (FC) and Herzberg-Teller (HT) contributions to electric-dipole transition moments including the displacements along all active vibrational modes. Here, the HT mechanism was found much more important; only *a_g_* and *b_1g_* modes produce intense lines in free-base porphyrin fluorescence [17], in agreement with polarization measurements [15,19]. Two weak wide bands observed in the gas phase absorption spectra of the H_2_P molecule at 626 and 576 nm could be interpreted as the 0-0 and 0–1 bands of the *^1^A_g_* → *^1^B_3u_* transition, respectively. The 0–1 band with largest contributions from the *ν_10_(a_g_)* = 1,610 cm^−1^ and *ν_19_(b_1g_)* = 1,600 cm^−1^ modes [17] was found to be in agreement with previous tentative assignments [15,19,20]. Both bands were found to include asymmetric stretching vibrations of the methine bridges [17]. A number of fine structure bands, including combination of two vibrational quanta, were obtained and compared with available site-selected spectra from Shpolskii and noble-gas matrices. Both absorption and fluorescence spectra could be interpreted on the basis of the linear coupling model and a good applicability of the mirror-symmetry rule was established [17].

Dendritic encapsulated metalloporphyrins mimic efficiently a number of functions expressed in biological systems. These are hemoglobin- and myoglobin-like gas-binding ability, heme mono-oxygenase activity, electron-acceptor capacity in light-harvesting antenna systems, and shell-modulated redox potentials as found in cytochromes [5]. One very interesting property of the dendritic molecules is their ability to create a microenvironment inside. Such dendron coating can protect porphyrins from the surrounding environment [21–25]. The site isolation can be used for protecting an active pigment photo-center from de-excitation by oxygen [23] or potentially even change monomolecular photophysical parameters, hence to some extent controlling the lifetimes of the excited states. Such controlled molecular photosystems could be of use for applications like optical power limiting devices [26,27] or in sensing applications [28]. For such and related purposes, porphyrins decorated with bis-MPA dendrons were prepared [29]. Specifically, Bis-MPA (2,2-bis(methyolol)propionic acid) repeating units were used as building block in the synthesis of dendron-coated meso-tetraphenyl porphyrins (TPP). They were further functionalized both as free-base porphyrin (TPPH_2_) and with a central zinc ion (TPPZn). Different sizes of molecules in terms of a systematic variation of the size of the dendrimer substituent were prepared, and their basic properties investigated [29]. For example, the hydrodynamic volume of the dendrimers could be determined from polarization anisotropy decay data, and it was established that the bis-MPA dendrimers are significantly smaller than the same generation Fréchet-type [30] benzyl ether TPP dendrimer. The larger dendrimer substituents of the zinc ion case gave rise to entirely new features in the absorption and fluorescence spectra [29]: A broad shoulder at longer wavelengths was more prominent in the emission spectra of the larger dendrimers however, only in the case with the zinc ion in the center. The proto-porphyrin analogue did not show this size-effect. Since a large substituent could impose a larger “stress” to the molecule than a small one, we anticipate that this could also affect the porphyrin ring configuration and its associated vibronic structure to different extent.

**Figure 1.**
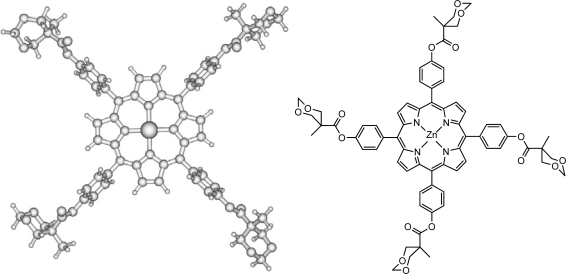
Model structure for the dendron substituted tetraphenyl Zn-porphyrin (TPPZn) molecule used in the calculations.

Here, the results of more detailed photophysical studies are presented along with results of a detailed theoretical investigation of the vibronic structures relevant for the interpretation of the electronic spectra. The H_2_P molecule is in essence the heart of all porphyrins and calculations of its detailed vibronic structure [17] were used as a guide-line for analysis of absorption and fluorescence spectra of meso-tetraphenyl derivatives and bis-MPA dendrimers grown on the basis of para-substituted tetraphenyl porphyrins. Specifically, we calculated the infrared (IR), absorption and non-resonance Raman spectra of the parent molecules HO-TPPH_2_, HO-TPPZn and by inference use the results to discuss results of dendrimers based on acetonide-2,2-bis(methoxy)propanoic (bis-MPA). The vibrational spectra are interpreted on the basis of density functional theory with the B3LYP functional [31] and different basis sets together with our previous studies of vibrations in H_2_P and ZnP molecules [16,17]. IR and Raman spectra of free-base *meso*-tetraphenyl porphyrin (TPPH_2_) and TPPZn are also calculated and compared with published data [6,20,32–37]. Most previous IR and Raman spectroscopy studies of porphyrins were performed using substituted derivatives because of their high solubility and easier access. Detailed vibration spectra of the parent molecules, H_2_P and ZnP, have been experimentally and theoretically studied quite recently [7,38–40] however, some old assignments of tetraphenyl derivatives [32–35,37] are still controversial. We used DFT calculations for all these molecules in order to make a consistent interpretation of IR, Raman, electronic absorption and fluorescence spectra of bis-MPA dendrimers, and a model compound used in the calculations is shown in Figure 1, to be further discussed in the results and discussions section.

## 2. Results and Discussion

### 2.1. General Appearance of Porphyrin Optical Absorption Spectra

**Figure 2.**
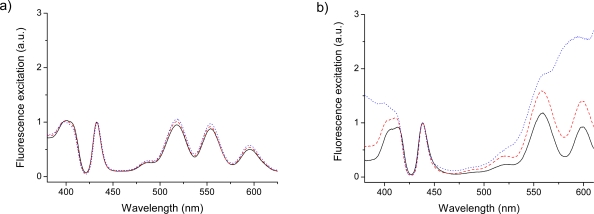
Flourescence excitation spectra of the G0 (solid), G2 (dash) and G4 (dotted) variants of dendrimer capped TPP. (a) Acetonide-G_x_-prop-TPPH_2_ for emission at 650 nm. (b) Acetonide-G_x_-prop-TPPZn for emission at 660 nm.

As follows from Figure 2 in Vestberg *et al*. [29], all optical absorption spectra of dendrimers are quite typical for porphyrins but include some additional features specific for the dendrimer substituted prophyrins. For the sake of discussion, representative steady state fluorescence excitation spectra for a number of TPPZn and TPPH_2_ dendrimers are shown in Figure 2. In order to interpret the dendrimer peculiarities one needs to comment on the common features of porphyrin chromophores. The first excited singlet state of the H_2_P molecule is *^1^B_3u_* and the same “effective” symmetry can be used for the tetraphenyl derivative, since the electronic excitation is located mostly in the porphyrin ring (we use the common choice of axes [16]: the x-axis coincides with the N-H bonds, the z-axis is perpendicular to the plane of the molecule). This gives the *Q_x_* weak absorption band. For the H_2_P molecule it consists of two peaks, at 626 and 576 nm, which are interpreted [15,17,19] as the 0-0 band of the *^1^A_g_* → 1*^1^B_3u_* transition and the 1-0 band, respectively.

As follows from DFT vibronic calculations [16,17] and from high-resolution Shpolskii spectra [15,19] both bands consist of a number of different vibration modes; thus the interval between two maxima has nothing in common with a particular single vibration. The latter band has the largest contributions from the *ν_10_(a_g_)* = 1610 cm^−1^ and *ν_19_(b_1g_)* = 1600 cm^−1^ modes (see notations of vibration modes in [20]). In TPPH_2_ and in all of its dendrimer variants these two peaks are at 652 (this band does not show up in the excitation spectra of Figure 2a) and 595 nm, thus indicating a red shift. The second weak *Q_y_* band of the H_2_P molecule also consists of two peaks at 510 nm (0-0) and 480 nm (1-0); the latter peak is more intense [50]. These are showing up at 518 and 554 nm in the excitation spectra (Figure 2a). Both 1-0 bands borrow intensity from the Soret band, which is produced by close lying 2*^1^B_3u_* and 2*^1^B_2u_* excited states. These *Q_x_* and *Q_y_* bands in free-base porphyrin are interpreted in terms of the well-known four orbitals model [50]. Our DFT calculation results supported this model and also reproduce the red and blue shifts in the derivatives (Table 1).

The vibronic 1-0 transitions of the Q bands are more intense than the 0-0 transitions in the absorption spectra of all simple porphyrins [50], but this is not the case for tetraphenyl derivatives. The main difference between emission properties of free-base porphyrins and Zn-porphyrins is connected with the longer radiative lifetime of the former [50]. In tetraphenyl derivatives (TPPH_2_ and TPPZn) and in dendrimers the 0-0 band is much more intense in fluorescence than the red-shifted 0–1 band [29]. The dendrimer variants of TPPZn also follow the general picture as long as the dendrimer substitutions are small. As reported for the optical absorption spectra and fluorescence the larger dendrimers (notably G4 and G5) gives entirely different spectra. This is also noted for the excitation spectra of the G4-dendrimer, as shown in Figure 2b. In order to understand these and other vibronic features in time-resolved fluorescence spectra of dendrimers it is necessary to first study their vibrational frequencies from the IR and Raman spectra.

### 2.2. IR Spectra of TPPH_2_, TPPZn and Porphyrin Dendrimers

**Figure 3.**
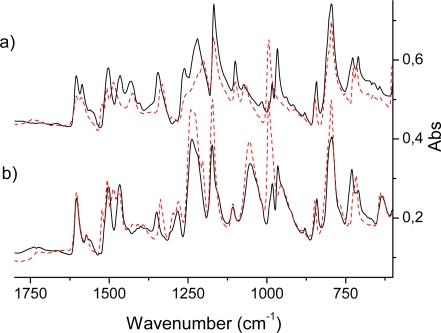
(a) Infrared absorption spectra of the HO-TPPH_2_ (solid) and HO-TPPZn (dashed) molecules. (b) Infrared absorption spectra of the HO-prop-TPPH_2_ (solid) and HO-prop-TPPZn (dashed) molecules. N.b., the spectra of panel a) was added a constant (0.25) in order to make the plot.

**Figure 4.**
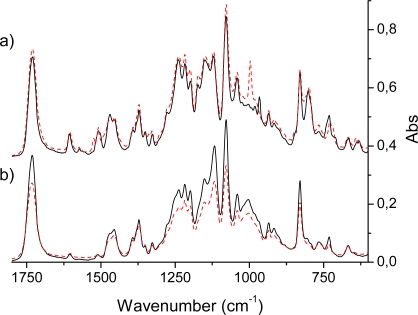
(a) Infrared absorption spectra of Acetonide-G2-prop-TPPH_2_ (solid) and Acetonide-G2-prop-TPPZn (dashed) molecules. (b) Infrared absorption spectra of Acetonide-G5-prop-TPPH_2_ (solid) and Acetonide-G5-prop-TPPZn (dashed) molecules. N.b., the spectra of panel a) was added a constant (0.3) in order to make the plot.

Representative IR spectra of the free-base and Zn porphyrin dendrimers are displayed in Figures 3 and 4. IR spectra of tetraphenyl porphyrins have previously been studied in a number of works and the assignment of several IR bands has been proposed [32–34,37,54]. The band near 1,600 cm^−1^ was interpreted as a C-C vibration of the phenyl substituents; its shift upon deuteration supported this assignment [32]. The low-frequency region was studied by Kincaid and Nakamoto [33]; isotopes of different metal-ions revealed the modes at 400–470 cm^−1^ which include metal vibrations.

In order to get a consistent description of all tetraphenyl derivatives at the DFT level we need to start to discuss the vibrational assignment of simple porphyrins. Firstly, we compared our B3LYP/6-31G** calculated IR absorption spectra of H_2_P and ZnP molecules, which previously were studied and interpreted by empirical force-field [20,54] and quantum scaled force-field calculations [7,38,55]. The H_2_P molecule belongs to the *D_2h_* point group and has 108 vibrational modes, which can be separated into in-plane (73) and out-of-plane (35) modes. The former vibrations can be classified in the H_2_P molecule as belonging to 19 *a_g_* + 18 *b_1g_* + 18 *b_2u_* + 18 *b_3u_* modes. The *b_2u_* and *b_3u_* vibrations of the H_2_P molecule are transformed into degenerate *e_u_* modes in the ZnP molecule which belongs to the *D_4h_* point group. These modes are active in IR spectra together with the out-of-plane porphyrin ring vibrations of *b_1u_* (H_2_P) and *a_2u_* (ZnP) symmetry. The correlation of vibration modes in H_2_P and ZnP molecules is summarized in Table 2.

The number of out-of-plane vibrational modes in H_2_P can be divided into the symmetry classes 8 *b_3g_* + 9 *b_2g_* + 8 *a_u_* + 10 *b_1u_*. The former two symmetry types are allowed to occur in the Raman spectrum, but they are not very active as follows from our calculations and previous results [20,38,56]. In ZnP there are 105 fundamental vibrations which have the following distribution in the symmetry classes belonging to the *D_4h_* point group, 71 in-plane vibrations: 18 *e_u_* + 9 *a_1g_* + 9 *b_1g_* + 8 *a_2g_* + 9 *b_2g_*, 34 out-of-plane vibrations: 8 *e_g_* + 3 *a_1u_* + 3 *e_u_* + 5 *b_1u_* + 4 *b_2u_*. (N.b., vibrations of *e_u_* and *e_g_* symmetry are doubly degenerate). The B3LYP DFT/6-31G** and /3-21G methods were employed in order to establish correlation between IR spectra of dendrimers and their generic ancestors. Though tetraphenyl derivatives and dendrimers are non-planar the use of the *D_2h_* and *D_4h_* symmetry point group notations is still useful, since the electronic features and force fields of the simple tetrapyrrole rings are mainly responsible for the UV and IR spectra of the dendrimers. Assignments of the most intense IR and Raman bands in the ancestors of dendrimers are presented in Tables 3–4, and Tables 5–6, respectively. Since the low-frequency part of the IR spectra was not available in our experimental data the comparison of the theoretical analysis will focus on the intense experimental absorption in the 600–1800 cm^−1^ region. Thus we have excluded C-H and N-H stretching with high frequencies (more than 3000 cm^−1^) from our Tables. Their spectral assignments are trivial [7,38,55].

#### 

##### IR active intense out-of-plane porphyrin ring vibrations

An intense IR absorption starts to grow at 700 cm^−1^ and gives the first very strong band at 785 cm^−1^ in the H_2_P molecule [16,20,54,55] by excitation of the vibrational mode ν_43_ (following our throughout numeration of Table 3) of *b_1u_* symmetry. This is an out-of-plane wagging vibration of the N-H and C-H bonds in the protonated pyrrole rings with weak involvement of the C*_m_*-H bonds (C*_m_* are the methylene-bridge carbons). Because of the substitution in the TPPH_2_ and HO-TPPH_2_ molecules this mode is slightly shifted being mixed with the phenyl C-H bending. For the tetraphenyl derivatives in the same region there are also four close-lying intense lines determined by pure out-of-plane symmetric C-H bending in phenyl rings (δ*_CH_*Ph). By overlap with the porphyrin mode ν_43_ they give one of the most intense lines at 790 cm^−1^ in the TPPH_2_ and HO-TPPH_2_ molecules (Figure 3). In the ZnP molecule this vibration corresponds to the ν_38_ mode of *a_2u_* symmetry (Table 4). It consists of C-H out-of-plane wagging for C*_β_*-H bonds and includes also the C*_m_*-H wagging vibrations (out-of-phase to the former). Since the nature of this mode is rather different in the H_2_P and ZnP molecules (no N-H bond in the latter) the frequency of the ν_38_ mode is shifted in ZnP to 765 cm^−1^ and the corresponding intensity decreased (Table 4). The experimental frequency shift for this mode, n_H2P_-n_ZnP_ = 20 cm^−1^, can be compared with the calculated one (29 cm^−1^). In TPPZn [37] and HO-TPPZn (Figure 3) this line is also overlapped by four intense δ*_CH_*Ph bands with frequencies 795 and 797 cm^−1^, respectively; resulting in a larger shift compared to ZnP (in comparison with free-base variants) because of the stronger involvement of the C*_m_*-H wagging vibrations. It is also more strongly mixed with the C-H bending vibrations of the phenyl rings. In the HO-prop variants (Figure 3b) the intensity ratio for the free-base and Zn porphyrins is reversed in agreement with DFT/3-21G calculations. In dendrimers this IR band is more shifted to 802 cm^−1^ and a new close lying intense band 829 cm^−1^ occurs (Figure 4). From the 3-21G calculation of the acetonide-G1-TPPZn dendrimer (Figure 1) the latter band is connected with a few CH_2_ modes of acetonide groups. The band 802 cm^−1^ is the former ν_43_ *b_1u_* mode of free-base porphyrin ring (Table 3) mixed with the δ*_CH_*Ph vibrations and with the C-O-C bending in the acetonide groups. Its intensity is diminished for the dendrimer model in agreement with calculation; there are also a number of close lying new (acetonide) lines. A similar behaviour of this IR band was observed for Zn-containing dendrimers (Figure 4).

The other intense line in the IR spectrum of the H_2_P molecule [38,55] at 852 cm^−1^ also belongs to the out-of-plane vibration of the *b_1u_* symmetry (ν_48_ in Table 3, mostly the C*_m_*-H bending) that slightly involves the C*_β_*-H bond vibration. Since this mode is strongly affected by meso-tetrathenyl substitution of the porphyrin ring, it is less intense and shifted to 842 cm^−1^ in TPPH_2_ and HO-TPPH_2_ molecules (Figure 3). The shift and intensity reduction are supported by our calculations. In ZnP this mode appears similar. It corresponds to the ν_49_ vibration of *a_2u_* symmetry (Table 4). Its measured frequency (849 cm^−1^) is almost the same as for H_2_P. The calculated frequencies and intensities are also very similar (Table 3 and Table 4).

The *b_1u_* out-of-plane vibrations of H_2_P correlate with the *a_2u_* and *b_2u_* symmetry of the ZnP molecule of the *D_4h_* point group (Table 2) and only the former vibrations are IR active. Although the optimized TPPZn and HO-TPPZn molecular structures are nonplanar, we can use correlation with the *D_4h_* symmetry since some intense vibrations are determined by characteristic modes of the porphyrin ring. Since the ν_48_ is one of the dominating out-of-plane vibrations of the C*_m_*-H bonds, it is quite natural that the corresponding intensity is strongly reduced upon tetraphenyl substitution (Figure 3). This mode is transformed in TPPZn in such a way that it includes out-of-plane vibrations of phenyl rings. In dendrimer substituted TPPs this vibration is quenched; the corresponding out-of-plane vibrations is shifted to the low-frequency region (the vibrations of light H atoms are transformed into out-of-plane movement of the massive and bulky substituent). It should be noted that the out-of-plane vibrations were not considered in empirical force-field calculations [20,54] and we need to use our throughout numeration of all modes, as presented in Tables 3 and 4. The presented B3LYP/6-31G** calculations are in good agreement with the scaled results of Pulay *et al*. [7,38,55] with respect to intensity and polarization of IR and Raman spectra (Tables 3–6). Correlation with the AM1 results is strightforfard and obvious.

##### IR active in-plane porphyrin ring vibrations

There are no *b_1u_* (*a_2u_*) out-of-plane vibrations in the H_2_P (ZnP) molecules with frequencies larger than 854 cm^−1^ (Tables 3 and 4). The same was found in calculations of the porphyrin ring out-of-plane vibrations in the tetraphenyl derivatives. Thus, the remainder of the porphyrin IR spectrum is caused by in-plane vibrations, for which assignment and numeration of Li and Zhierski [20] are available. (Here, this numeration is denoted by a prime symbol ν′*_i_* in order to avoid confusion with our complete numeration including also out-of-plane vibrations.) Analyses of the in-plane vibrations were also reported in a large number of experimental studies [32–34,37,54,57]. We introduce the discussion of the vibrational structure with the ZnP analysis since it is more convenient to consider first the degenerate *e_u_* vibrations, and then to discuss the corresponding vibrations of lower symmetry that occur in e.g., dendrimer capped porphyrins.

The most prominent features in the IR spectrum of the ZnP molecule [20] occur at 993 and 1,052 cm^−1^ and originate from the *e_u_* vibrations (ν*_54,55_* and ν*_62,63_*, respectively, Table 4). The former mode includes the out-of-phase C_α_-C_β_ stretching vibrations in the opposite-lying pyrrole rings with the corresponding Zn-N asymmetric stretches. This is the most intense (doubly degenerate) vibrational transition of the IR spectrum of ZnP (Table 4) in agreement with experiment [7]. In the notation of Ref. [20] this is the ν′*_47_* mode, described as a pyrrole breathing mode. The results of our DFT calculations are not in complete agreement with the interpretation of Ref. [20]. Our results also contradict the assignment of the 993 cm^−1^ mode suggested in Ref. [57]. The weaker line at 1,052 cm^−1^ originates from C_β_-H deformations being out-of-phase in the opposite-lying pyrrole rings. Both these modes (993 and 1,052 cm^−1^) are mixed with the phenyl vibrations in TPPZn. Because of the specific character of the ν*_54,55_* vibrations, the C*_m_*-Ph stretches are silent in TPPZn and the frequency 993 cm^−1^ is hardly shifted in the tetraphenyl derivatives, TPPZn [37], HO-TPPZn and HO-prop-TPPZn (Figure 3). The mode at 993 cm^−1^ of ZnP is split in the TPPZn molecule into two close lying frequencies (992 and 994 cm^−1^ in our scaled DFT calculation), both having relatively high intensity (61 and 65 km/mole). They also include C-C vibrations of the phenyl rings. In the dendrimers, intensity of these very characteristic vibrations of the porphyrin ring are significantly changed. In the acetonide-G2-prop-TPPZn dendrimer the line at 993 cm^−1^ is not the most intense and slightly shifted to 996.8 cm^−1^. Its intensity is reduced by 25%, since the bulky acetonide groups withdraw electron density from the porphyrin ring and quench the dipole moment derivative along this C_α_-C_β_ stretching vibration. In the acetonide-G5-prop-TPPZn dendrimer, the intensity of this line is reduced much more (by 67%) for similar reasons; the band is broadened because of mixing with acetonide vibrations and the maximum is shifted to 1002 cm^−1^. At the same time the weaker line 1,052 cm^−1^ of ZnP changes considerably upon tetraphenyl substitution, since these modes ν*_62,63_*, directly involve C*_m_*-H bending modes. In the the TPPZn and HO-TPPZn molecules this frequency shifts to 1,069 and 1,067 cm^−1^, respectively. In HO-prop-TPPZn it is overlapped by an intense band at 1,055 cm^−1^ (Figure 3) originating from O-(CH_2_)_3_ vibrations. This intense IR absorption in dendrimers is further increased and shifted to 1,080 cm^−1^ (Figure 4). The line at 1,052 cm^−1^ of ZnP is shifted approximately to 1,041 cm^−1^ in the dendrimers. Since this C_β_-H bending also involves some C*_m_*-Ph bending character, its frequency is sensitive to substituents in the phenyl rings of TPPZn and is reduced in the more flexible bis-MPA dendrimers. The other *e_u_* vibrations of low intensity in the IR spectrum of ZnP are not so informative as the intense lines mentioned above and we omit their discussion.

In free-base porphyrin and its derivatives the corresponding *e_u_* vibrations of ZnP are split in the *D_2h_* point group of the H_2_P molecule into *b_2u_* and *b_3u_* modes [7,20]. The ZnP mode at 993 cm^−1^ (ν*_54,55_*, Table 4) is split into 951 cm^−1^ *b_2u_* and 971 cm^−1^ *b_3u_* modes in H_2_P (ν*_53_* and ν*_55_*, in Table 3). The nature of these modes is the same as in ZnP (asymmetric breathing of the opposite-lying pyrrole rings), but the absence of the Zn-ion and Zn-N stretches releases the force constants and leads to low-frequency shifts. The *b_2u_* mode shifts more since it corresponds to unprotonated pyrrole rings. These two lines are not so intense in H_2_P, like the double-degenerate line at 993 cm^−1^ of the ZnP molecule (Tables 3 and 4). Instead of the intense ZnP peak in this region, there is a gap (weaker absorption) in the IR spectrum of the H_2_P molecule and this is the main difference between the two spectra. This trend is also well observed for IR spectra of the HO-TPPZn and HO-TPPH_2_ molecules (Figure 3), but not in the dendrimers (Figure 4), since the line at 993 cm^−1^ is not the most intense in acetonide-Zn-prop-TPPZn derivatives, as discussed above. The line corresponding to 993 cm^−1^ of HO-TPPZn splits in the HO-TPPH_2_ molecule into 983 and 966 cm^−1^ lines (analogous the the case of *b_3u_* and *b_2u_* modes, respectively; Figure 3). As far as the ZnP infrared line 1,052 cm^−1^ is concerned, the behavior of free-base porphyrin variants is very peculiar; it splits into the 1,043 cm^−1^ and 1,054 cm^−1^ bands. They correspond to our ν*_61_* (*b_3u_*) and ν*_62_* (*b_2u_*) modes, respectively (Table 3). In the H_2_P molecule they become C_β_- C_β_ -H bending vibrations of the out-of-phase type with respect to the opposite pyrrole rings. The ν*_61_* (*b_3u_*) vibration involves the protonated rings, and the ν*_62_* (*b_2u_*) mode involves the unprotonated rings (Table 3). Only the ν*_61_* (*b_3u_*) vibration is mixed with the C*_m_*-Ph bendings and only this mode is seen in IR absorption of dendrimers. The striking difference of ZnP and H_2_P vibrations of the C_β_-H type has not been stressed before, as will be discussed more below, it is important for our further analysis of the dendrimers.

The separated strong line at 1,731–1,733 cm^−1^ of all dendrimer samples is definitely connected with the carbonyl groups stretching. A very strong and narrow IR band at 1,080 cm^−1^ in the region of intense porphyrin absorption was also present in all dendrimer samples. This originated from acetonide vibrations mixed with porphyrin modes. Even though the band is narrow, it consists of few close lying intense lines of similar nature. They include wagging vibrations of CH_2_ and CH_3_ groups, deformation of the Ph-O-CH chain and ν*_61_* - ν*_63_* modes of the porphyrin ring (Table 3). The band 1,172 cm^−1^ of HO-TPPX and HO-prop-TPPX molecules corresponds to the single bond C-O stretching of the terminal COH groups. It disappears in dendrimers because there are no such groups in the acetonide moiety. The C-O-H bending vibrations are assigned to the strong line at about 1220-1240 cm^−1^ in the IR spectra of the HO-TTPX and HO-prop-TPPX molecules (X= Zn, H_2_P). The less intense, closely lying bands at about 1,260–1,280 cm^−1^ correspond to C-C-H bending vibrations of the phenyl rings; in the dendrimers they are shifted to lower frequency and overlapped by absorption of acetonide groups. The line at 1,349 cm^−1^ of HO-TTPH_2_ corresponds to the C*_m_*-Phenyl stretching vibrations; it is shifted to 1,369 cm^−1^ in dendrimers because of mixing with acetonide vibrations. The bands near 1,600 cm^−1^ at the edge of the HO-TPPX infrared spectra belong to the phenyl C=C vibrations; these are sensitive to substituents and are strongly reduced in the dendrimers because of admixture of the ether-group stretching. In HO-TPPX in the region 1,500-1,420 cm^−1^, there are a few intense IR bands of C-C stretching and C-C-H bending vibrations of the phenyl rings; some of them are mixed with C*_m_*-C*_α_* asymmetric modes of the porphyrin core. Introduction of the acetylide moieties leads to significant distortion of these bands. The broad strong IR absorption bands at 3,244 cm^−1^ (HO-TTPZn) and 3,219 cm^−1^ (HO-TTPH_2_), data not shown, are attributed to O-H stretching vibrations in agreement with the calculated results. They further split into four modes of *a_g_* and *b_u_* symmetry; the two latter *b_u_* modes correspond to paired combinations of O-H stretches from the opposite sides of the porphyrin ring. In the HO-prop-TPPX molecules these O-H stretching vibrational modes are shifted to higher frequency (about 3,320 cm^−1^) because of the inductive effect of the alkyl groups.

### 2.3. Raman Active Modes

The *gerade* modes which are active in Raman spectra are important for analysis of vibronic bands in dendrimer fluorescence, since they induce mixing between the *Q* and Soret states. Thus they provide the Hertzberg-Teller (HT) contribution to fluorescence intensity from the second term of Equation 1 (Methodology section). The totally symmetric vibrations are mostly important for the Franck-Condon (FC) terms. Because of the symmetry reduction in dendrimers some acetonide modes are simultaneously active in IR and Raman spectra, and in order to understand and interpret their occurrence in the fluorescence vibronic sub-structure it is necessary to analyse first the Raman activity of the porphyrin core.

#### 

##### In-plane vibrations active in Raman spectra

The resonance Raman (RR) spectra of tetraphenyl complexes with metals (TPPM) were previously assigned using a normal coordinate analysis of biphenyl with regard to assignment of phenyl modes [37,58]. In our normal coordinate analysis of tetraphenyl complexes the full Hessian obtained from DFT calculations was used. Taking the TPPZn molecule as an example one gets 225 real frequencies. In the low-frequency region the most intense line of the Raman spectrum of the H_2_P molecule is the mode ν_13_ =309 cm^−1^ of *a_g_* symmetry (Table 5; thiscorresponds to ν′_8_ in the numeration of [20]). This corresponds to C_α_-C*_m_*-C_α_ in-phase bending vibrations and hindered translation of all pyrrole rings and can also be described as a uniform breathing of the whole tetrapyrrole ring [20,38]. In ZnP it is shifted to 363 cm^−1^ (ν_18_ in Table 6). In TPPZn it is detected at 387 cm^−1^ [37,58]. This vibration was also seen in the fluorescence spectrum taken using a low-temperature solid matrix [14].

It is well known that the RR spectra of the TPP derivatives are usually dominated by the porphyrin skeletal modes due to a resonant effect, although some phenyl modes have also been observed indicating evidence for π-delocalization to phenyl rings [35]. Resonance enhancement of the Raman scattering occurs only if the vibrational mode involves atoms which are part of the electronically excited chromophore. The dihedral angle between the porphyrin ring and meso-phenyl substituent planes is close to 70° from our DFT optimization, being in general agreement with experimental data (80°) [35]; thus the π-systems of the porphyrin and phenyl rings should not interact. At the same time the RR spectrum of TPPZn has a strong band at 1,236 cm^−1^ that has been assigned as the C*_m_*-Ph mode [37]. From our DFT analysis it is mixed with the internal phenyl C-C stretches. The Raman intensity of this and other phenyl modes can be explained in terms of hyperconjugation. In fact the LUMO *e_g_* orbital of TPPZn has large π-expansion coefficients at the C*_m_* atoms; at the same time it has appreciable admixtures of *2s*-orbitals at the ortho-carbon atoms of the phenyl rings. Thus the π-σ hyperconjugation occurs upon the π-π* excitation, explaining the Raman activity of the phenyl modes. This could also be observed in the fluorescence spectrum of low-temperature solid TPPZn as the onset of the blue wing of the 0–1 band at 650 nm [14].

The maximum of the 0–1 band in porphyrins is determined by two asymmetric C*_α_*-C*_m_* stretching vibration modes [17]. In the H_2_P molecule these modes are very close in frequency (1610 and 1600 cm^1^; ν_94_ and ν_92_ in Table 5), belonging to *a_g_* and *b_1g_* symmetry, respectively. These are ν′_10_ and ν′_19_ modes in notations of Ref. [20]. The former RR line is very intense, the latter one is weak. In ZnP molecule these two vibrational frequencies are separated by about 50 cm^−1^ (These are the ν_93_ and ν_91_ modes in Table 6). The interaction between adjacent C_α_-C*_m_* and C_α_-N bonds in ZnP have much larger negative effect on the ν′_19_ mode than on the ν′_10_ mode, as also was pointed out in Ref. [20]. In tetraphenyl porphyrins the ν′_19_ mode is shifted down by about 25 cm^−1^, being more in agreement to the results of our DFT calculations. This is because of its mixture with C_α_-C*_m_*-C*_phenyl_* vibrations. Our scaled prediction for the TPPZn molecule (ν′_19_ = 1,545 cm^−1^) is in a good agreement with the resonance Raman frequency measurement [37] (1,548 cm^−1^). It was natural to propose that a large shift of the ν′_19_ frequency in ZnP in comparison with the H_2_P can be responsible for the difference in their fluorescense spectra [17].

The next less intense line in H_2_P fluorescence is ν′_20_ = 1,388 cm^−1^ of *b_1g_* symmetry [17,20] (vibrational mode ν_79_ in Table 5). In ZnP it has slightly lower frequency (1353 cm^−1^) and belongs to *a_2g_* symmetry [20]. In the *D_4h_* point group this mode and the close lying ν′_26_ vibration are non-active in the Raman spectra. The interpretation of these closely lying vibrations of *a_2g_* symmetry (ν′_20_ and ν′_26_) in the ZnP molecule is very important for analysis of fluorescence vibronic bands (*a_2g_ x E_u_ = E_u_*; thus, these modes are active in mixing of the states giving the characteristic *Q* and Soret bands). In our DFT study (Table 6) they correspond to modes number 75 and 76, respectively. The former includes asymmetric C*_m_*-H stretches accompanied with strong deformations of the pyrrole rings (mostly C_α_-N- C_α_ asymmetric stretching). The ν′_26_ = 1,322 cm^−1^ mode [20] corresponds to our number 75 in Table 6; including both asymmetric C*_β_*-H and C*_m_*-H stretches. Thus, it is expected that in tetraphenyl porphyrins the ν′_26_ mode is shifted down by about 90 cm^−1^. In TPPZn we calculated it to be 1,233 cm^−1^. Similar results were obtained for the H_2_P and the TPPH_2_ molecules. Accounting the results of vibronic calculations [17], it is here suggested that the ν′_26_ = 1,237 cm^−1^ (*a_2g_*) mode contributes to the formation of the 0–1 band in the fluoresecnce spectrum of the HO-TPPH2 molecule with a wide maximum at about 1200 cm^−1^. The ν′_26_ mode includes the C*_m_*-C*_phenyl_* stretching and according to our calculations it is responsible for effective mixing between the *Q* and Soret states.

In the fluorescence spectra of the TPPH_2_ molecules the 0-0 band (658 nm) is much more intense than the 0–1 band (714 nm) because the phenyl substituents are not in the porphyrin plane. This deviation from planarity and from the *D_2h_* symmetry provides an increase of the electronic 0-0 transition moment of the *Q_x_* band. The quantum yield of fluorescence is also increased upon tetra phenyl substitution of the H_2_P molecule [50]. Thus the 0-0 line is more intense than all 0–1 lines because of the stronger Franck-Condon mechanism in comparison with the Herzberg-Teller mechanism for borrowing intensity [17]. The energy gap between the 0-0 and 0–1 bands in the TPPH_2_ molecule in benzene [50] is 1,410 cm^−1^. This gap depends on the solvent: using a mixture of ethyl-iodide the gap was found to be 1,538 cm^−1^ [50]. For H_2_P the gap is largest [17]: 1,620 cm^−1^. This solvent and substituent dependence of the frequency separation between the 0-0 and 0–1 bands in fluorescence of porphyrins has never been explained so far. It is here suggested that it can be interpreted as the result of more involvement of the ν′_20_ and ν′_26_ modes of *b_1g_* symmetry in H_2_P upon tetraphenyl substitution. The ν′_10_ (*a_g_*) and ν′_26_ (*b_1g_*) modes at about 1,600 cm^−1^, which correspond to the C_α_-C*_m_* asymmetric stretching vibrations, produce the most intense 0–1 vibronic line in the H_2_P molecule and are strongly reduced upon tetraphenyl substitution. The massive phenyl groups are naturally admixed into these vibrations and contribute some 60 cm^−1^ down-shift; more important is a reduction of vibronic mixing and of the corresponding 0–1 lines intensity in fluorescence and *Q_x_* band absorption. Keeping in mind these peculiarities of the ν′_10_, ν′_19_ and ν′_26_ modes we now can progress by considering the absorption and fluorescence spectra of porphyrin-cored bis-MPA TPP dendrimers.

### 2.4. Interpretation of Optical Absorption Spectra

We have to point out that at first glance there are no large differences observed in the absorption and fluorescence spectra of dendrimers of different generations [29]. This agrees with earlier findings for tetraphenyl porphyrin dendrimers of Frechet type [59]. The spectra show the typical absorption bands of porphyrins (Soret-band and *Q*-bands) and the difference in the *Q*-bands between free base and zinc-containing porphyrins can clearly be seen [29]. This difference is well-reproduced in TD DFT calculations of the singlet-singlet absorption spectra of the H_2_P and ZnP molecules [12,16,60] and in their tetraphenyl derivatives (Table 1). The metal porphyrins are characterised by a blue shift of the *Q*-band with respect to the free bases. For the ZnP and H_2_P molecules the calculated shift is 0.18 eV; whereas the experimental shift is 0.2 eV (Table 1). For tetraphenyl porphyrins there are red shifts with respect to simple porphyrins: for TPPZn it is 0.14 eV (0.09 eV), for TPPH_2_ the red shift is equal to 0.12 eV (0.11 eV); experimental data shown in parentheses. The absorption spectra for both free base and zinc porphyrin dendrimers in THF were depicted in Figure 2 of Vestberg *et al*. [29].

The calculated red shift of the *Q*-band between TPPH_2_ and HO-TPP_2_ molecules is very small (0.0087 eV, or 2 nm in wavelength) which agrees well with spectral measurement in dichloromethane [61]. Further substitution in para-position of the phenyl rings by OCH_3_ and OCH_2_CH_2_CH_3_ groups provides no shift. This partially explains why no apparent shifts in the *Q*-bands maxima between the dendrimer generations are observed. The four weak absorption bands of acetonide-Gn-prop-TPPH2 dendrimers at 649, 592, 552 and 514 nm [29] can be interpreted as the *Q_x_*(0-0), *Q_x_*(1-0), *Q_y_*(0-0) and *Q_y_* (1-0) bands in free-base tetraphenyl porphyrin moiety, respectively, and the peaks apart from the long wavelength absorption are also shown up in fluorescence excitation spectra when monitoring the emission at 660 nm, as shown in Figure 2a. The spectrum is similar to the absorption spectrum of the TPPH_2_ molecule in the same THF solvent [50,62]. In Zn-porphyrins there is only one degenerate (1^1^*E_u_*) excited state in this region (Table 1) which is responsible for the *Q*(0-0) and *Q*(1-0) absorption bands at 600 and 558 nm, respectively.

With more detailed analysis the absorption spectra of acetonide-G0/G5-prop-TPPH2 and acetonide-G0/G5-prop-TPPZn dendrimers indicate some differences in both classes. In the case of free-base porphyrins, increased absorption just above 450 nm was observed for the fifth generation. This absorption is also present for the higher-generation zinc-cored porphyrin, where it is much more pronounced. One possible reason for this could be connected with the rise of the red wing of the Soret band (shown in Figure 2 of Ref. [29]). But this explanation is not supported by the fluorescence excitation spectra of the dendrimers (Figure 2a). From our calculations (Table 1) it follows that there are forbidden transitions to the *^1^B_1g_* states in this region. For ZnP molecule they correlate with the *^1^A_2g_* and *^1^B_2g_* states that correspond to π-π* (4*e_g_* – 5*e_g_*) transitions with large “metal-to-ligand” charge-transfer character. The MO 4*e_g_* has about 30 % of the metal 3*d_π_*-orbital contribution; MO 5*e_g_* is a double degenerate LUMO of the porphyrin ring. Transition to the *^1^A_2g_* state is rather peculiar since it is the only one that has a large magnetic dipole transition moment (μ_z_ = 1.9 *μ_B_*, where *μ_B_* is the Bohr magneton). In the TPPZn molecule this transition is red-shifted by 8.3 nm with respect to ZnP following our DFT/3-21G calculation; besides its magnetic-dipole character it acquires electric dipole transition moment (*D_z_* = 0.01 *ea_0_*, where *a_0_* is the Bohr radius).

The LUMO of all porphyrins has large contributions from the C*_m_* atoms. In ZnTPP it is connected by hyperconjugation with the 2s-orbitals at the ortho-carbons of the phenyl rings. Because of the hyperconjugation with phenyl rings the *gerade* symmetry of the porphine chromophore is removed and the former 4*e_g_* – 5*e_g_* transition becomes electric-dipole allowed. The analogous *^1^A_2g_* state of the HO-TPPZn molecule is further red-shifted by 9.4 nm and the *X^1^A_1g_* – *^1^A_2g_* transition is greatly enhanced. The nature of the transition is changed; here it includes large contribution of charge transfer from π-orbitals of the phenyl rings (oxygen atoms are also included). Substitution in para-position of the phenyl rings by OCH_3_ groups leads to further increase of the transition moment. One possible explanation could be that the dendrons also interact with the phenyl rings, making these transitions more allowed. The stronger absorption to vibrational levels of the *^1^A_2g_* and *^1^B_2g_* states giving the substructure at 450–500 nm could be enhanced by the heavy dendron substitution. This absorption is more enhanced in metal-porphyrin dendrimers, which can be the subject of the Jahn-Teller effect [52]. This is readily observed in the absorption spectra of Ref. [29], as well as in the excitation spectra of the larger dendrimer substitions, when monitoring the emission at 650 nm, Figure 2b. Additional interesting feature of the absorption spectra for the zinc-containing porphyrins is the appearance of a weak shoulder at around 630 nm. This red absorption was clearly observed for the second generation and becomes more pronounced for the larger substituents. We suppose that this is an indication of the Jahn-Teller splitting of the 1^1^*E_u_* state in ZnP upon tetraphenyl substitution (Table 1) and its enhancement in dendrimers.

At this point it is necessary to consider connection with the so-called “hyperporphyrin” spectra [36,50,63]. Hyperporphyrins have been defined as porphyrins that exibits extra absorption bands in the region λ > 320 nm that are not of the π−π* nature of the tetrapyrrole ring [50]. These extra bands are proposed to be due to charge transfer (CT) interactions between the tertrapyrrole ring and either metal or substituents. Washing the porphyrin-stained laboratory glassware with acid one can see clear manifestation of the hyperporphyrin spectroscopy; acid turns a reddish tetraphenylporphyrin stain into brilliant green [63]. At the same time porphyrins without tetraphenyl substituents do not undergo such visual change on acidification. The reason is that the protonated TPPH_2_ molecule is a hyperporphyrins [63]. In acid solvents the diprotonation occurs and the *Q_x_* and *Q_y_* bands are transformed into one *Q*-band red-shifted by 30–140 nm, depending on phenyl-substituent [36,63]. Such a strong sensitivity to the substituent in the para-phenyl position is important not only for the dication but also (to less extent) for the neutral species. The molecules studied in the present work are not hyperphorphyrins, but the observed small changes in the absorption spectra can be explained by the similar trends found in the tertraphenyl hyperphorphyrins.

To the blue side of the *Q*(1-0) absorption band (555 nm) of the Zn containing dendrimers there are growing features at about 530 nm [29]. They also are seen in Frechet-type dendrimers [59]. In the excitation spectra of the largest dendrimers this increase is even more pronounced as the vibration sublevels collapses into a band at approx. 580 nm. In accord with our B3LYP/3-21G TD FDT calculations this absorption can be interpreted as an enhanced σ(3d_x2−y2_) – π* transition. In the ZnP molecule this is a forbidden transition (10*b_1g_* - 5*e_g_*) to the 1^1^*E_g_* state, which is overlapped by the *Q*-band (Table 1). In H_2_P there is no metal and no such transition (the σ − π* transition of similar symmetry in the H_2_P molecule is much higher in energy and is overlapped by the Soret band, Table I). In the TPPZn and HO-TPPZn molecules the σ(3d_x2−y2_) – π* transition is electric dipole allowed and successively enhanced because of the hyperconjugation of the porphyrin LUMO with tetraphenyl rings and dendrons. The calculated oscillator strength of this transition in TPPZn is very small (3.3 × 10^−5^) but in the HO-TPPZn molecule the oscillator strength of the σ(3d_x2−y2_) – π* transition increases by approx. 50%. This effect was examined by changing conformations of a series of acetonide-G1-TPPZn dendrimers by imposing asymmetric distortions to the substituents followed by geometry optimization/relaxation. Although many isomers have essentially the same total energy certain such isomers resulted in that the transition into the ^1^*E_g_* state is splitted and produces two lines (typically, 517 and 511 nm following from the B3LYP/3-21G TD calculation) with a common oscillator strength increased to 0.0029. For other isomers with more symmetric arrangement of the dendron groups the splitting still exists (515 and 512 nm) but with reduced transition intensity. Thus, only for dendrimers the σ(3d_x2−y2_) – π* transition became observed. This prediction was obtained only with the 3-21G basis set; the 6-31G** basis set predicts the 1*^1^E_g_* state at higher energy and such interpretation of weak absorption at 530 nm and in the 580 nm regions is not definite.

Calculations of electronic excited states by employing the B3LYP/3-21G TD FDT method indicated that the addition of the OH-group provides a small red-shift of the *Q* band (73 cm^−1^, or 2 nm) in HO-TPPZn in comparison with the TPPZn molecule. But for the Soret band of the HO-TPPZn molecule the DFT method predicts a stronger red-shift in comparison with TPPZn in agreement with observations. The reason is connected with the larger involvement of charge transfer from the phenyl rings to those excitations, as determined in the four-orbital scheme. The position of the Soret band in the acetonide-G0/G5-prop-TPPZn dendrimers is slightly shifted to the red side (2–4 nm) upon increase of the dendrimer generation. In free-base analogs, where the Jahn-Teller effect is absent, the shift is negligible. Even the *Q*(0-0) absorption band of the acetonide-G5-prop-TPPZn dendrimer is blue-shifted as a result of the Jahn-Teller splitting of the 1^1^E_u_ state. The difference between free-base and zinc-containing dendrimers even is more apparent in fluorescence spectra as will be discussed in the following section.

### 2.5. Fluorescence of Porphyrin bis-MPA Dendrimers

**Figure 5.**
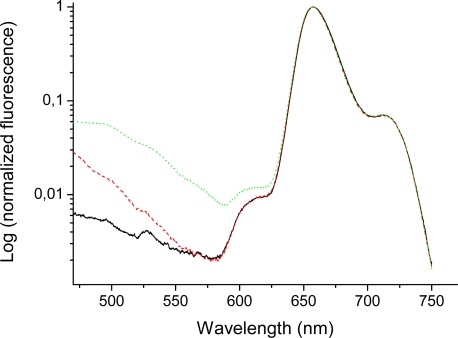
Emission spectra (logarithmic scale) of Acetonide-G0-prop-TPPH_2_ (solid/black), Acetonide-G3-prop-TPPH (dashed/red), and Acetonide-G5-prop-TPPH (dotted/green). Concentration 0.010 mM (THF); excitation wavelenth 403 nm. Slits 5 and 10 nm for excitation and emission, respectively.

**Figure 6.**
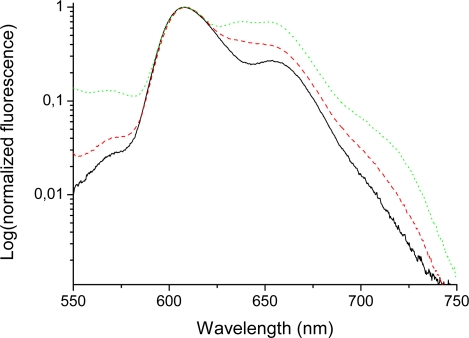
Emission spectra (logarithmic scale) of Acetonide-G0-prop-TPPZn (solid/black), Acetonide-G4-prop-TPPZn (dashed/red), and Acetonide-G5-prop-TPPZn (dotted/green). Concentration 0.010 mM (THF); excitation wavelenth 403 nm. Slits 5 and 10 nm for excitation and emission, respectively.

The emission spectra of bis-MPA coated porphyrins (in THF) in the long wavelength region (600–750 nm) (Figure 5 and 6) indicate quite large differences for higher generations of the HO-prop-TPPZn dendrimers in comparison with the dendron coated free-base variant. The emission of the free-base porphyrin dendrimers shows two peaks, one strong peak at 658 nm and a weaker peak at 714 nm, similar to TPPH_2_ in different solvents [50,62]. The former peak corresponds to the 0-0 transition and the latter one to the 0–1 transition. These vibrational frequencies are in the range 1100–1300 cm^−1^. Moreover, there is no difference in the emission spectra of different generations of the free-base coated dendrimers in this long wavelength region (Figure 5). All free-base porphin-cored bis-MPA dendrimers have the same 0-0 and 0–1 bands in the emission spectra, however, for the zinc-cored porphyrins the spectra show a dramatic different behavior in the region of *Q*(0-0) and *Q*(0–1) bands (600–750 nm). The spectrum shows a strong peak at 610 nm, which corresponds to the *Q*(0-0) band and a smaller one (for Acetonide-G0-prop-TPPZn) around 650 nm, the *Q*(0–1) band. As can be seen in Figure 6, the emission around 650 nm increases with increasing generation (the difference is largest between the fourth and the fifth generation [29]; in the latter case a distinct new band at 637 nm appears). The growth of new emission at long wavelengths in TPPZn dendrimers is connected with increased vibronic interaction between porphyrin core and acetonide groups induced by the mixed modes, which touch simultaneousely the ring distortion with the Zn atom in the middle and deformation of phenyl-prop-acetonide moiety.

**Figure 7.**
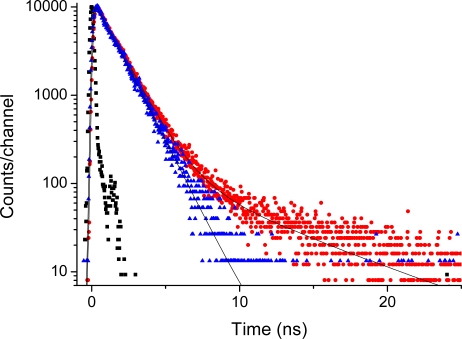
Emission decay of Acetonide-G2-prop-TPPZn (0.050 mM). Red dots: Excited at 403 nm with emission monitored at 650 nm. Blue triangles: Excited at 403 nm with emission monitored at 600 nm. Black squares is system response used for de-convolution fit; slit 32 nm.

**Figure 8.**
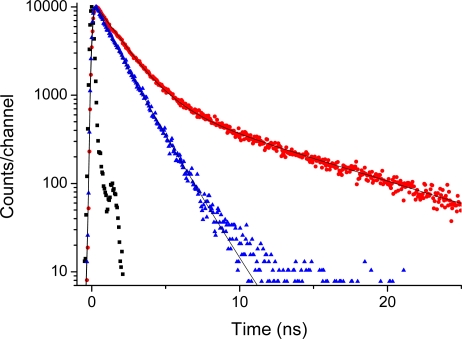
Emission decay of Acetonide-G5-prop-TPPZn (0.050 mM). Red dots: Excited at 403 nm with emission monitored at 650 nm. Blue triangles: Excited at 403 nm with emission monitored at 600 nm. Black squares is system response used for de-convolution fit; slit 32 nm.

This is supported by the time-resolved emission data presented in Figures 7 and 8. The time-decays shown in Figures 7 and 8 are for Acetonide-G2-prop-TPPZn, and the G5 dendrimer, respectively. The molecular systems, here dissolved in THF, were excited at 403 nm and the emission at 650 nm is coded with red dots, whereas the emission at 610 nm is coded with blue triangles. The emission at 650 nm corresponds to the broad vibronic shoulder in the emission spectrum (Figure 6). With no dendrimer, or the smallest dendrimer, the decay-trace can be analysed using a single time-constant of 1.3 - 1.5 ns. This holds true for all dendrimers when detecting the emission at 610 nm. For the larger dendrimers there is an additional contribution from a considerably slower component for the emission at 650. This is readily observed as a “kink” and a decay of slower time-constant in Figure 8. The decay time-constant of this slower decay was between 6.5 and 9 ns (generally, decay times are longer for both the fast and slow components with increasing dendrimer size). Notably, the relative contribution of slow decay in respect to the fast component is larger the larger the dendrimer substituent, so it changes from approximately 5/100 for the G1 dendrimer, up to 35/100 for the G5 dendrimer. There was no change in decay times by varying the dye concentration between 5 and 100 micromolar. Hence, we can attribute this slow component to a true intrinsic “molecular” feature and not to an aggregation effect. The emission decay was also recorded for the series of dendrimer capped TPPH_2_ variants however, here there was no essential difference between the various dendrimers (data not shown). For the whole series the decay trace could in general be analysed with one dominating (single) decay constant being approximately 9 - 10 ns. Hence, the substituent (dendron) size-effect in the long wavelength region (600–750 nm) of fluorescence emission was exclusive for only the case of dendrimer coated TPPZn.

The decay time of all dendron coated free-base variants was in the same order of magnitude as the “slow” component growing in with increased substituents in the TPPZn case. Thus, a plausible reason for appearance of this new emission could be contamination of free base porphyrin in the zinc porphyrin samples since the growing peak occurs in the region of the free base porphyrin fluoresecnce (655 nm). However, no free-base porphyrin was detected in the NMR and UV spectra obtained for the zinc porphyrins [29]. Another explanation could be that the larger, and notably the fifth-generation, dendrimer was not perfect leading to unsymmetrical substitution of the porphyrin. However, this explanation seems not likely since no traces of such asymmetric units could be observed for the free base variant that was produced using the same procedure. This was also emphasized by the size-exclusion chromatography and the results of other measurements previously reported [29]. Notably, the different dendrimer substituted TPPH_2_-cases showed very similar decay traces but a pronounced difference was observed and measured for the time-resolved anisotropy decay, allowing an analysis of their hydrodynamic volume [29]. Taken together, both photo-physical measurements along with other chemical purification and characterization leads to exclusion of “contamination” effects as a cause of the dendron size effect observed for the substituted TPPZn variants.

We hereby return to the discussions of these results in more detail, in particular the heavy substitution effect observed for the TPPHZn dendrimers. The explanation for this could be interactions between the porphyrin and the dendrons changing the porphyrin conformation and vibrational substructure. Similar changes upon dendron substitution were observed in the absorption spectra as discussed above. The peak at 610 nm in absorption of Zn-containing dendrimers, which is the 0-0 transition to the lowest singlet excited state, corresponds to the 1^1^*E_u_* state of ZnP (Table 1). The excited degenerate state is a subject of the Jahn-Teller distortion and it splits into 1^1^*B_3u_* and 1^1^*B_2u_* states upon tetraphenyl substitution even at the ground state optimized geometry (Table 1), which in fact is lower than *D_2h_*. The nonplanar tetraphenyl substituents do not perturb the degeneracy very much, thus one can speak about the pseudo-Jahn-Teller effect. At this point it is important to mention that the lowest vibrational 0-level in the distorted state is the same in all dendrimer generations. The pseudo-Jahn-Teller effect is calculated by geometry optimization in the excited state of the HO-TPPZn molecule and it is found stronger than in zinc-porphyrin.

The occurence of strong and new peaks to the red-side of the 0,0 band in emission of the Acetonide-G5-prop-TPPZn dendrimer we can explain by manifestation of the pseudo-Jahn-Teller effect in the lowest excited singlet state. Though the *D_4h_* symmetry is reduced in TPPZn, in HO-TPPZn and in dendrimers, the quasi-degeneracy of the 1^1^*E_u_* states is still present. The pseudo-Jahn-Teller effect is induced by distortion along the *b_1g_* active mode as follows from our geometry optimization in the excited singlet state by employing the TD B3LYP method. The ground state *D_4h_* symmetry is reduced to the *D_2h_* symmetry in the excited state of the ZnP molecule. The *b_1g_* active mode corresponds to the low-frequency vibration (ν_7_ in Ref [16]) in the ground state of ZnP molecule. This is a Zn-N stretching and the corresponding translation of all pyrrole rings. A similar analysis was applied for the TPPZn molecule, though it belongs to the *C_2_* point group, since the quasidegeneracy still exists (Table 1). In this case the Zn-N stretching is mixed with the out-of-plane deformations of the C-H bonds in the phenyl rings. In the model of Acetonide-G1-TPPZn dendrimer (Figure 1), calculated with 3-21G basis set, this mode is mixed with the twist and rock vibrations of the methyl groups. The Jahn-Teller effect cannot occur in the molecules of free-base porphyrin type and in their dendrimers; this can explain the large differences in fluorescence spectra of the dendron coated TPPH_2_- and TPPZn-species (Figures 5 – 8, Ref [29]).

The vibrational modes connected with phenyl vibrations (frequencies in the range 750–830 cm^−1^) are becoming more active with increasing generation of TPPZn dendrimers along with the increase of the Jahn-Teller effect. We optimized the HO-TPPHZn molecular geometry in the excited singlet state by the TD B3LYP/3-21G method. The C-OH bond length in the hydroxy groups decreases upon excitation in agreement with the known fact that phenol is a stronger acid in the excited state than in the ground state [36]. The decrease of the C-OH bond length leads to stronger energy shift of the *Q*-state (the stronger Jahn-Teller effect) and to an increase of the *S_0_* *- S_1_* transition moment. At the same time the transition moment becomes more sensitive to phenyl vibrations in the range 750–830 cm^−1^. All these findings can explain the increase of vibronic bands in fluorescence of Acetonide-Gx-prop-TPPZn dendrimers at 650 nm and shorter wavelength for higher generations (Figures 6 and 8).

There are many conformers of dendrimers. For a model shown in Figure 1 we have found more than 20 species by molecular mechanics, but the possible number is much larger. There are six conformers of TPPH_2_ which are very close in energy (in the range of 1.4 kcal/mol) [64] and the number increases to 144 for HO-TPPH_2_. Being close in the ground state energy they have different frequencies for some vibrational modes of the terminal groups. We can estimate that the number of close-energy conformers of Acetonide-G5-prop-TPPZn dendrimer is larger than a thousand. All of them are present in solvent at room temperature and provide slightly different wavelengths of vibronic transitions. This explains some broadening of vibronic bands in absorption, excitation and emission spectra (Ref. [29], Figure 6 and 8).

To the blue side from the 0-0 peak in the fluorescence spectra of all dendrimers there are number of growing emission bands (Figures 5 and 6) which are determined by forbidden transitions from the 1^1^*B_3g_*,1^1^*B_2g_* excited states (Table 1) strongly influenced by substitution at para-position of phenyl rings. Here we just want to stress that this emission from the highly excited states (550 nm) was not detected before in porphyrins, as far as we know (we do not consider the blue fluorescence from the Soret state, which is induced at 430 nm by strong laser impulse [65]). The forbidden transitions to the 1^1^*B_3g_*, 1^1^*B_2g_* states (Table 1) are observed in absorption spectra of dendrimers (Figure 2 from Ref. [29]) and in fluorescence excitation spectra in Figure 5. These states have been discussed recently with respect to the MgP and ZnP spectra, calculated at different geometries of the porphyrin core [60]. In free-base porphyrin they have much higher energy than in Zn porphyrins (Table 1; they are of σ − π* nature in PH_2_ and 3d_σ_-π* nature in Zn-porphyrin). Their energy strongly depends on the *b_1g_* distortion discussed above, indicating the Jahn-Teller effect in Zn-porphyrin. These states are important for calculation of metal-porphyrin phosphorescence since their triplet counterparts are mixed with the ground singlet state by spin-orbit coupling [60]. As it was mentioned above this *^1^E_g_*-state in TPPZn dendrimers is responsible for absorption at 520 nm and is enhanced in higher generations. The other *gerade* states (1^1^*B_1g_*, 2^1^*A_g_* in PH_2_ and 1^1^*B_2g_*, 2^1^*A_2g_*, 1^1^*B_1g_* in Zn-porphyrin, Table 1) are of the π − π* nature. They have been considered to be responsible for two-photon absorption in TPPH_2_ [49]. The energy of those states are close to the Soret band origin; their vibronic transitions probably contribute to the weak emission of dendrimers below 500 nm.

## 3. Methodology

### 3.1. Sample Preparation

The synthesis and characterization of dendron-coated porphyrins up to the fifth generation were previously described in [29]. The porphyrin used was 5,10,15,20-tetrakis(4-hydroxyphenyl)-21H-,23H-porphyrin. Both free-base and zinc cored tetraphenylporphyrin (TPPH_2_ and TPPZn) were designed with dendrimer coatings. From the porphyrin the dendrons were divergently grown using the anhydride of acetonide protected bis-MPA (acetonide-2,2-bis(methoxy)propanoic anhydride). Synthetic procedure schemes, structures and other properties are discussed by Vestberg *et al*. [29].

### 3.2. Spectroscopy

The Fourier transform infrared absorption spectra were collected by employing a Perkin-Elmer Spectrum 2000 FT-IR equipped with a MKII Golden Gate, Single Reflection ATR System from Specac Ltd, London. The ATR-crystal was a MKII heated Diamond 45° ATR Top Plate. 16 scans were recorded for each spectrum. Time-resolved fluorescence decays were recorded using an IBH 5000 U fluorescence lifetime spectrometer system wth a TBX-04 picosecond photon detection module. The emission monochromator resolution was 1 nm. IBH NanoLED-10 (443 nm) and NanoLED-07 (405 nm) were used as excitation source for decay measurements of single photon excitation. Melles Griot colored glass filter was used to block scattered light from the excitation source. The fluorescence lifetime decays were measured using time-correlated single photon counting (TC-SPC) along with the IBH Data Station v2.1 software for operation of the spectrometer and deconvolution and analysis of decays. The DAQ-card settings were chosen to give a time resolution below 10 ps. Steady state absorption and fluoresence spectra of bis-MPA dendrimers were presented in Figures 2 and 3 of Vestberg *et al*. [29] For the sake of discussion we present excitation spectra (corresponding absorption spectra for selected emissions) as well as fluorescence spectra of both acetonide-G0/G5-prop-TPPH_2_ and acetonide-G0/G5-prop-TPPZn types of dendrimers, the latter in logarithmic scale to provide a better comparison of spectral intensities. The steady state emission is complemented with novel time-resolved data recorded at different emission wavelengths in order to aid in the interpretation.

### 3.3. Theory

Very few large molecules have attracted such attention among theorists as porphyrins. The early theoretical studies were limited to semiempirical methods [41] which explained the main features of the electronic absorption optical spectra, but required some adjustable parameters. Later, a number of *ab initio* methods [9,42–44] and DFT calculations [10–12,16,45–47] were applied in order to explore their electronic properties. Nonetheless, in spite of a large amount of experimental and theoretical data, there are still many unknowns regarding the structural, electronic, and optical properties for various porphyrins, and many fine details remain to be elucidated [48,49]. One of these puzzles is the 0–1 vibronic band in tetraphenyl porphyrins spectra. In H_2_P and ZnP molecules it is more intense than the 0-0 band [13,17,50] while in tetraphenyl derivatives the 0-0 band is more intense, especially in emission spectra.

In the absorption and fluorescence spectra of HO-TPPH_2_ and HO-TPPZn molecules and their bis-MPA dendrimers presented in Figures 2–3 of Vestberg *et al*. [29] we found conspicuous differences in the vibronic structure of the 0–1 bands of free-base and zinc porphyrins including bis-MPA dendrimers. These became more prominent with higher generations of the Bis-MPA dendrimers attached to the porphyrin ring. In order to interpret these differences one has to take into account both electronic and vibrational states. The general expansion of the total wave function in the adiabatic Born-Oppenheimer approximation Y*_i,n_*(*q,Q*)=F*_i_*(*q,Q*)c *_n_*(*Q*) can be used, where F*_i_* is the electronic *i*-state wave function, *q* and *Q* denote electronic and nuclear coordinates, respectively [51]. In the harmonic approximation the nuclear wave function c*_n_*(*Q*) is a product of wave functions of all harmonic vibrational modes *Q_a_*. The 0-n vibronic Ψ_*i*,0_ → Ψ*_f,n_* transition intensity is determined by the transition dipole moment [51,52]

(1) $$\langle \Psi_{i,0}|q+Q|\Psi_{f,n}\rangle =M_{i,f}\;{\left(Q\right)}_{0}\langle \chi_{0}|\chi_{n}\rangle +\sum_{a}\frac{\partial M_{i,f(Q)}}{\partial Q_{a}}\langle \chi_{0}|Q_{a}|\chi_{n}\rangle$$

where

(2) $$M_{i,f}\;(Q)=\langle F_{i}\;(q,Q)\;|q|\;F_{f}\;(q,Q)\rangle$$

The first term corresponds to the Franck-Condon (FC) contribution, the second term corresponds to the Herzberg-Teller (HT) contribution of the vibronic spectrum. The FC mechanism arises from the difference in equilibrium geometry between the ground and excited states. The HT contribution results from vibronic coupling between excited electronic states. Similar types of FC and HT mechanisms occur in vibronic theory of Raman spectra [51].

We used direct calculation of the total energy first and second derivatives in respect to atomic displacements by the DFT [18]. Vibrational modes are obtained by diagonalization of the Hessian; IR and Raman intensities are calculated by numerical differentiation of the dipole moment and polarizability, rescpectively. FC factors are estimated through the gradients [52]. We use the linear vibronic coupling model [52,53] recently implemented in the time-domain DFT approach for calculation of free-base porphyrin spectra [16,17].

## 4. Conclusions

The synthesis and basic properties of dendrimers based on free-base tetraphenylporphyrin and zinc-tetraphenylporphyrin (TPPH_2_ and TPPZn) were reported some years ago [29]. These porphyrins were coated with the acetonide-2,2-bis(methoxy)propanoic anhydride dendrons and preliminary results of their photo-physical properties were reported [29]. The molecules were further studied by IR and fluorescence spectroscopy as presented herein. Theoretical simulations of these spectra employing density functional theory (DFT) calculations indicated some new assignments in vibrational spectra of related porphyrins and in dendrimers. Account of vibronic interactions in simple porphyrins leads us to conclude that the pseudo-Jahn-Teller effect induced by distortion along the *b_1g_* active mode in the quasi-degenerate states of the 1*^1^E_u_* type is responsible for the intensity increase of wide vibronic bands in the fluorescence of dendron substituted TPPZn variants. The strong vibronic interaction leads to increased Jahn-Teller distortion of the prophyrin core at higher dendrimer generations. Specifically, this is manifested as an entirely different behaviour of the emission spectra upon substitution of the TPPH_2_ and TPPZn variants. The 0–1 emission band of the dendron substituted TPPZn experiences a “heavy substitution” effect, giving rise to a 0–1 emission signal associated with a longer decay time (7 - 8 ns) than for the 0-0 emission (1 - 1.5 ns). This contributes with stronger relative emission yield for larger dendron substituents, also in agreement with the appearance of steady state emission spectra showing increased contribution from the 0–1 emission at 650 nm. Since TPPH_2_ is originally of lower symmetry, the specific distortion upon dendron substitution is not expected, and this was also in agreement with the experimental findings.

## Figures and Tables

**Table 1.** Electronic absorption spectrum of the free-base porphyrin, Zn-porphyrin, and their tetraphenyl derivatives. Excitation energy is in eV, oscillator strength (f) is given in parentheses. Experimental gas-phase data are from ref [13].

**H_2_P**			**TPPH_2_**		**ZnP**			**TPPZn**	

D2h	6-31G**	Exp.^a^	6-31G**	Exp.^a^	D4h	6-31G**	Exp.^a^	6-31G**	Exp.^a^
1^3^B_2u_	1.46	1.56	1.39	1.50	1^3^E_u_	1.74	1.82	1.70	1.61
1^3^B_3u_	1.81	1.87	1.69	1.70	1^3^E_u_	1.74	1.82	1.70	1.61
1^1^B_3u_	2.27(10^−4^)	1.98(0.01)	2.15(0.03)	1.87(0.08)	1^1^E_u_	2.45(10^−3^)	2.18(0.04)	2.31(0.03)	2.09(0.04)
1^1^B_2u_	2.44(10^−3^)	2.42(0.06)	2.15(0.03)	1.87(0.08)	1^1^E_u_	2.45(10^−3^)	2.18(0.04)	2.31(0.03)	2.09(0.04)
1^1^B_2g_	3.76(0.00)	-	3.74(0.00)	-	1^1^E_g_	3.33(0.00)	-	3.29(0.01)	-
1^1^B_3g_	3.86(0.00)	-	3.85(0.00)	-	1^1^E_g_	3.33(0.00)	-	3.29(0.01)	-
2^1^B_3u_	3.33(0.41)	3.33(0.56)	3.16(0.85)	3.08(0.70)	2^1^E_u_	3.49(0.90)	3.22(0.70)	3.24(0.67)	3.05(0.70)
2^1^B_2u_	3.41(0.60)	3.33(0.56)	3.21(0.85)	3.08(0.70)	2^1^E_u_	3.49(0.90)	3.22(0.70)	3.26(0.81)	3.05(0.70)
1^1^B_1g_	3.39(0.00)	-	3.38(0.00)	-	1^1^A_2g_	3.41(0.00)	-	3.43(0.00)	-
1^1^B_1g_	3.50(0.00)	-	3.51(0.00)	-	1^1^B_2g_	3.64(0.00)	-	3.55(0.00)	-
3^1^B_2u_	3.56(0.45)	3.50(0.05)	3.82(0.10)	3.80(0.06)	3^1^E_u_	3.80(0.04)	3.82(0.05)	3.45(0.08)	3.57
3^1^B_3u_	3.61(0.86)	3.50(0.05)	3.82(0.10)	3.80(0.06)	3^1^E_u_	3.80(0.04)	3.82(0.05)	3.47(0.09)	3.57
1^1^B_1u_	3.85(10^−3^)	3.65(<0.1)	3.82(0.01)	-	1^1^A_2u_	3.65(0.01)	-	3.60(0.02)	-
4^1^B_3u_	3.89(0.57)	-	4.12(0.24)	4.96(0.1)	4^1^E_u_	4.26(0.09)	-	4.02(0.06)	-
4^1^B_2u_	3.92(0.48)	-	5.37(0.09)	5.97(0.1)	4^1^E_u_	4.26(0.09)	-	4.05(0.07)	-
2^1^B_1u_	5.89(0.003)	-	5.82(0.01)	5.97(0.1)	2^1^A_2u_	5.01(0.08)	-	4.92(0.09)	-

**Table 2.** Correlation of vibrational symmetry between ZnP (*D_4h_*) and H_2_P (*D_2h_*) molecules.

**In-plane**		**Out-of-plane**	

ZnP	H_2_P	ZnP	H_2_P
*e_u_*	*b_2u_*	*e_g_*	*b_2g_*
*e_u_*	*b_3u_*	*e_g_*	*b_3g_*
*a_1g_*	*a_g_*	*a_1u_*	*a_u_*
*b_1g_*	*a_g_*	*b_1u_*	*a_u_*
*a_2g_*	*b_1g_*	*a_2u_*	*b_1u_*
*b_2g_*	*b_1g_*	*b_2u_*	*b_1u_*

**Table 3.** The part of the infra-red spectrum of free-base porphyrin in the region 760 – 1,750 cm^−1^. “Int.” is the IR absorption intensity (km/mol), “*ν_i_*“ the wavenumber (cm^−1^).

		6-31G** (this work)		Scaled 6-31G* Ref [38]		**Exp. Refs [54, 67]**

i	Sym	*ν_i_*	Int.	*ν_i_*	Int.	*ν_i_*
41	*b_1u_*	795.8	60.54	776.1	53.2	773
43	*b_1u_*	808.1	120.13	786.0	140.1	785
48	*b_1u_*	872.0	143.75	853.3	145.4	852
53	*b_2u_*	973.3	82.79	945.2	89.30	951
55	*b_3u_*	994.3	54.61	968.0	56.4	971
57	*b_2u_*	1011.7	10.41	981.5	5.7	977
59	*b_3u_*	1023.4	0.08	996.3	0.4	994
61	*b_3u_*	1079.4	43.58	1048.6	43.0	1043
62	*b_2u_*	1083.8	35.90	1053.0	35.7	1054
66	*b_3u_*	1172.9	20.62	1137.8	20.5	1134
67	*b_2u_*	1188.6	0.02	1155.8	0	1165
70	*b_3u_*	1231.7	2.82	1206.4	3.4	1177
72	*b_2u_*	1269.2	57.07	1225.4	57.8	1228
73	*b_2u_*	1282.9	0.78	1251.7	0.3	1255
74	*b_3u_*	1321.9	1.64	1286.3	1.8	1287
78	*b_2u_*	1393.7	4.41	1354.8	4.5	1357
81	*b_3u_*	1444.7	28.9	1407.6	26.0	1396
82	*b_3u_*	1448.7	4.00	1400.0	6.4	1412
83	*b_2u_*	1451.8	10.81	1409.3	10.3	1406
89	*b_3u_*	1570.4	7.03	1522.3	5.0	1522
90	*b_2u_*	1591.8	24.76	1546.8	22.6	1540
93	*b_2u_*	1644.8	17.23	1594.6	16.4	-

**Table 4.** The part of the infra-red spectrum of Zn-porphyrin calculated employing the B3LYP DFT method. “Int.” is the IR absorption intensity (km/mol), “*ν_i_*“ the wavenumber (cm^−1^).

		6-31G* Ref [7]		6-31G** This work		**Exp. Ref [7]**

i	*D_4h_*	*ν_i_*	Int.	*ν_i_*	Int.	*ν_i_*
37,36	*e_u_*	742	25.0	755	24.0	739
43,42	*e_u_*	799	3.3	808	5.0	799
54,55	*e_u_*	990	94.8	1016	95.0	993
59,60	*e_u_*	1020	0.9	1044	0.1	1019
62,63	*e_u_*	1055	56.8	1086	56.2	1052
67,68	*e_u_*	1153	1.0	1186	8.1	1151
71,72	*e_u_*	1252	0.1	1280	0.2	-
73,74	*e_u_*	1304	10.7	1342	15.3	1299
80,81	*e_u_*	1397	10.9	1427	6.3	1384
84,83	*e_u_*	1436	2.2	1482	3.0	1438
87,88	*e_u_*	1529	8.7	1572	9.2	1517
90,89	*e_u_*	1551	9.7	1603	7.5	1558
11	*a_2u_*	209	34.6	148	29.3	-
15	*a_2u_*	344	13.0	343	7.0	-
34	*a_2u_*	712	12.8	714	8.8	699
38	*a_2u_*	766	63.2	779	69	765
49	*a_2u_*	49	145	879	122	849

**Table 5.** The most important Raman fequencies of free-base porphyrin. “R” is the Raman scattering activity (Å^4^/amu), “*ν_i_*“ the wavenumber (cm^−1^).

		6-31G** This work		6-31G* Ref [38]		**Exp. Ref [19]**	**Exp. Ref [38, 54]**

i	*D_2h_*	*ν_i_*	R	*ν_i_*	R	*ν_i_*	*ν_i_*
4	*b_1g_*	98.9	17.2	86.9	16.9	-	109
7	*a_g_*	156.6	27.9	152.9	27.4	-	155
13	*a_g_*	310.4	74.8	303.8	77.0	-	309
18	*b_1g_*	395.9	0.10	388.4	0.2	-	389
19	*b_2g_*	420.1	1.29	410.2	1.0	-	418
30	*b_2g_*	712.9	9.89	700.3	9.9	-	-
31	*b_3g_*	714.6	11.3	700.3	11.5	-	-
32	*a_g_*	734.0	19.0	720.6	25.5	723	723
33	*a_g_*	738.5	12.5	727.8	9.4	-	736
54	*a_g_*	976.8	88.3	950.8	66.5	953	952
56	*b_1g_*	999.0	5.06	966.5	4.4	972	976
58	*a_g_*	1013.1	43.6	984.7	51.4	987	988
60	*b_1g_*	1028.5	8.59	1002.1	7.1	1005	1005
63	*a_g_*	1086.9	0.71	1055.8	0.6	1063	1063
64	*a_g_*	1093.7	6.13	1061.9	6.2	-	1064
65	*b_1g_*	1169.6	0.14	1133.2	0.1	-	1138
68	*a_g_*	1210.1	37.6	1179.3	36.2	1177	1177
69	*b_1g_*	1222.8	1.62	1186.3	1.6	-	1182
71	*b_1g_*	1261.9	2.46	1219.5	1.9	1221	1226
75	*b_1g_*	1357.3	55.7	1320.5	52.3	1316	1313
76	*a_g_*	1392.1	42.2	1358.1	45.5	1360	1353
77	*b_1g_*	1393.3	13.44	1354.7	17.4	1352	1374
79	*b_1g_*	1422.4	28.0	1381.1	31.3	1388	1388
80	*a_g_*	1442.2	224	1402.0	224	-	1384
84	*a_g_*	1477.7	110	1430.4	105	-	1425
86	*b_1g_*	1538.6	13.6	1491.2	15.3	1497	1493
87	*a_g_*	1551.1	297	1504.2	279	1502	1492
91	*a_g_*	1604.3	292	1558.7	280	1575	1544
92	*b_1g_*	1640.7	0.26	1590.3	0.40	1578	1600
94	*a_g_*	1654.8	203	1605.9	213	1614	1609

**Table 6.** The most important Raman frequencies of ZnP. “R” is the Raman scattering activity (Å^4^/amu), “*ν_i_*“ the wavenumber (cm^−1^).

		6-31G** This work		6-31G* Ref [7]		**Exp. Ref [7]**

i	*D_4h_*	*ν_i_*	R	*ν_i_*	R	*ν_i_*
7	*b_1g_*	177.6	23.2	177.2	23.7	-
9,10	*e_g_*	208.5	7.50	204.2	7.50	208.5
11	*b_2g_*	224.4	19.4	221.9	19.0	-
18	*a_1g_*	372.3	91.4	363.0	91.4	363
33,32	*e_g_*	715.4	10.8	707.6	11.5	-
34	*b_2g_*	738.6	6.25	726.5	24.5	728
56	*a_1g_*	1021.7	110.5	1001.4	102.4	995
65	*a_1g_*	1093.8	1.51	1063.4	38.3	1066
69	*a_g_*	1208.5	48.5	1182.4	39.1	-
70	*b_2g_*	1216.8	1.00	1181.6	0.4	-
75	*a_2g_*	1362.4	0.00	1329.5	0.00	1322
76	*a_2g_*	1388.7	0.00	1350.6	0.00	1353
77	*b_2g_*	1392.8	87.1	1357.2	86.2	1347
78	*a_1g_*	1399.9	39.3	1371.8	68.1	1357
79	*b_1g_*	1419.8	215	1387.3	202	1385
84	*a_1g_*	1485.1	111	1437.3	124	1432
86	*b_1g_*	1551.1	400	1503.6	378	1494
91	*a_1g_*	1603.2	289	1557.6	261	1544
93	*b_1g_*	1663.7	209	1612.6	229	1607
